# Total plasma cytochrome P450‐soluble epoxide hydrolase oxylipins, apolipoprotein E ε4 carrier status, and white matter hyperintensities in cerebrovascular disease

**DOI:** 10.1002/alz70856_107679

**Published:** 2026-01-09

**Authors:** William Z. Lin, Si Won Ryoo, Alexandra Magliocco, Natasha Z. Anita, Myuri Ruthirakuhan, Celine H.L. Huang, Sofia Perfetto, Douglas P. Munoz, Angela C. Roberts, Malcolm Binns, Anthony E. Lang, Sean Symons, Ameer Y. Taha, Walter Swardfager

**Affiliations:** ^1^ Sunnybrook Research Institute, Toronto, ON, Canada; ^2^ University of Toronto, Toronto, ON, Canada; ^3^ Queen's University, Kingston, ON, Canada; ^4^ Western University, London, ON, Canada; ^5^ Dalla Lana School of Public Health, University of Toronto, Toronto, ON, Canada; ^6^ Rotman Research Institute, Baycrest Academy for Research and Education, Toronto, ON, Canada; ^7^ Toronto Western Hospital, Toronto, ON, Canada; ^8^ University of California, Davis, Davis, CA, USA

## Abstract

**Background:**

The cytochrome P450‐soluble epoxide hydrolase (CYP450‐sEH) pathway has been implicated in cerebral small vessel disease (cSVD) due to the anti‐inflammatory and vasoactive effects of epoxide species produced by CYP450, which are converted into diols by sEH. However, the associations between total oxylipins and cSVD markers and the interactions with other risk factors (e.g., apolipoprotein E ε4 or *APOE* ε4 allele) remain unclear.

**Method:**

Participants diagnosed with cerebrovascular disease were identified from the Ontario Neurodegenerative Disease Research Initiative study. Periventricular and deep white matter hyperintensity (pWMH/dWMH) volumes at baseline were quantified using MRI images processed using a semi‐automated pipeline. 24 arachidonic, linoleic, eicosapentaenoic, and docosahexaenoic acid‐derived oxylipins were extracted from plasma with Folch extraction followed by hydrolysis, solid‐phase extraction, and quantification with LC‐MS/MS. Diol to epoxide ratios were calculated as a proxy for sEH activity. Cross‐sectional associations were examined using linear regression models between log‐transformed WMH volumes and log‐transformed oxylipin concentrations, including age, sex, diabetes, hypertension, creatinine, cholesterol, triglycerides, and presence of the *APOE* ε4 allele as covariates. Stratified analyses were performed within APOE ε4 carrier and non‐carrier subgroups.

**Result:**

Across 159 participants (67.9% male, mean age = 69.2 years), the eicosapentaenoic acid‐derived 11(12)‐epoxyeicosatetraenoic acid (EpETE) (β = 0.157, *p* = 0.034) and 14(15)‐EpETE (β = 0.155, *p* = 0.039) were positively associated with pWMH volumes. The 11,12‐dihydroxyeicosatetraenoic acid was also positively associated with pWMH volumes (β = 0.157, *p* = 0.031). The 9(10)‐linoleic acid diol/epoxide ratio was associated with greater dWMH volumes (β = 0.186, *p* = 0.031). In subgroup analyses, all omega‐3‐derived epoxides and diols were associated with greater pWMH volumes in *APOE* ε4 non‐carriers (β = 0.191 to 0.257, *p* = 0.027 to 0.002), whereas these effects were attenuated in *APOE* ε4 carriers. The 9(10)‐ and 12(13)‐linoleic acid diol/epoxide ratios were positively associated with dWMH volumes only in *APOE* ε4 non‐carriers.

**Conclusion:**

In individuals with cerebrovascular disease, *APOE* ε4 carrier status may moderate associations between CYP‐sEH oxylipins and WMH volumes. Studies of interventions targeting this pathway should consider stratification based on *APOE* genotype.

